# Healthcare Professionals’ Perspective on Implementing a Detector of Behavioural Disturbances in Long-Term Care Homes

**DOI:** 10.3390/ijerph18052720

**Published:** 2021-03-08

**Authors:** Mohamed-Amine Choukou, Sophia Mbabaali, Ryan East

**Affiliations:** 1 Department of Occupational Therapy, College of Rehabilitation Sciences, University of Manitoba, Winnipeg, MB R3E 0T6, Canada; mbabaal3@myumanitoba.ca (S.M.); eastr@myumanitoba.ca (R.E.); 2 Riverview Health Centre, Winnipeg, MB R3L 2P4, Canada; 3 Centre on Aging, University of Manitoba, Winnipeg, MB R3T 2N2, Canada

**Keywords:** dementia, behavioural disturbances, hazardous behaviour, safety, monitoring, privacy, trust

## Abstract

The number of Canadians with dementia is expected to rise to 674,000 in the years to come. Finding ways to monitor behavioural disturbance in patients with dementia (PwDs) is crucial. PwDs can unintentionally behave in ways that are harmful to them and the people around them, such as other residents or care providers. Current practice does not involve technology to monitor PwD behaviours. Events are reported randomly by nonstaff members or when a staff member notices the absence of a PwD from a scheduled event. This study aims to explore the potential of implementing a novel detector of behavioural disturbances (DBD) in long-term care homes by mapping the perceptions of healthcare professionals and family members about this technology. Qualitative information was gathered from a focus group involving eight healthcare professionals working in a tertiary care facility and a partner of a resident admitted in the same facility. Thematic analysis resulted in three themes: (A) the ability of the DBD to detect relevant dementia-related behavioural disturbances that are typical of PwD; (B) the characteristics of the DBD and clinical needs and preferences; (C) the integration of the DBD into daily routines. The results tend to confirm the adequacy of the DBD to the day-to-day needs for the detection of behavioural disturbances and hazardous behaviours. The DBD was considered to be useful and easy to use in the tertiary care facility examined in this study. The participants intend to use the DBD in the future, which means that it has a high degree of acceptance.

## 1. Introduction

### 1.1. Background

Dementia is a neurodegenerative disease commonly seen in older adults and characterized by fast-progressing cognitive impairments. The number of Canadians with dementia is expected to rise to 674,000 in the next decade [1]. There are currently over 1.1 million Canadians affected by dementia because they have a family member or friend living with dementia [2]. For instance, the number of Manitobans with Alzheimer’s disease or another form of dementia is expected to rise at an alarming rate, from 23,000 individuals currently to over 40,700 by 2038. The total economic burden of dementia in Manitoba is over 1 billion dollars and is expected to rise to over 28 billion by the year 2038 [1]. Patients with dementia (PwDs) experience mood swings [3], sleep problems [4], and behavioural issues [3,5] that make them emotionally frail. A strong emotional response to a minor problem is a common symptom for PwDs, in particular Alzheimer’s disease. Behavioural disturbances are defined in this study as events that are unintentionally committed by PwDs that may be harmful to themselves or others: i.e., falling, aimless wandering, hitting (including self), kicking, grabbing onto people, pushing, throwing things, biting, trying to leave the building, and tearing things or destroying property.

In PwDs, emotional frailty may mainly be due to anxiety. In mild cognitive impairment and Alzheimer’s disease, the majority of patients report anxiety symptoms [6,7]. Anxiety is a commonly observed symptom in patients with multiple neurodegenerative diseases, with prevalence rates situated between 8% and 71% for anxiety symptoms and between 5% and 21% for anxiety disorders [8]. For example, common signs of anxiety in Alzheimer’s patients include worried appearance, restlessness, agitation, fidgeting, and fearfulness [9]. Given the great variation in the different stimuli that can cause anxiety and the length and severity of the anxiety response per se, the commonality of all anxiety disorders is the dysregulation of fear [10]. In regular functioning, fear is a very useful emotional reflex that occurs naturally in response to threats, mobilizing the physiological resources needed (e.g., autonomic, somatic) and shielding us from danger [11]. However, when anxiety is not justified (i.e., arises in response to nonthreatening signals), it can be debilitating, cause excessive physiological stimulation and interfere drastically with normal functioning [11]. It is crucial to find solutions to monitor PwDs’ behavioural disturbances (e.g., due to anxiety) that could be harmful to themselves and to the people around them, such as other patients, staff, or visitors. Getting lost when outdoors greatly raises the risk of being involved in risky circumstances or even death [12,13].

Cognitive function affects mobility level. For instance, patients with severe dementia have significantly less mobility than patients with moderate dementia [14]. Those with better executive function have higher life-space mobility, which is more likely related to better walking capacity and the absence of transportation difficulties [15]. Life-space mobility has been correlated with comorbidity and complex health conditions, namely, hospitalization [16], cognitive function [17,18], falls [19], muscle atrophy [20], faecal incontinence [21], oral-health-related quality of life (QoL) [22,23], self-reported exhaustion [24] and frailty [25]. Other studies have documented associations between life-space assessment and contextual factors, namely, the physical environment and social network characteristics [26,27,28]. QoL decreases with reduced life space and limited cognitive and physical activity. Therefore, it is important to ensure continuous possibilities for safe out-of-room mobility in institutionalized PwDs to maintain their QoL. Life space is apprehended as a holistic measure of mobility behaviour to evaluate the enacted mobility behaviour, which includes the adaptation of the individual’s intrinsic capacity to the external environment’s constraints [28]. Life space is a crucial indicator of cognitive and physical health in individuals living in the community, describing the use of space where they move [27]. Literature has shown that characteristics and quality of life space affect cognitive and physical functioning (e.g., gait speed and balance [29] and dependence in the basic and instrumental activities of daily living [27,30,31]) and psychological mechanisms (e.g., depression [32], fear of falling [33] and perception of control [34]). Therefore, the paradigm of life space can be transposed to assess both mobility and behavioural changes in PwDs to inform holistic disease management strategies. The key determinants of mobility in this study include cognitive, psychosocial and physical environments. Behavioural keys include personal and interpersonal behaviours (e.g., agitation and fighting with other residents, respectively). Life-space evaluations are either long to perform for persons living in the community (e.g., [35]), meaningless in the context of long-term care home (LTCH), or subjective (e.g., [36]). In the current study, PwDs’ behavioural disturbances are studied from the perspective of posture and body language, which will be quantified using a versatile platform that is free of effort for staff and that provides objective data about behavioural issues that require “immediate” intervention.

In LTCH, events are reported randomly by nonstaff members or when a staff member notices the absence of a PwD from a scheduled event. The use of technology to monitor life space has been shown to increase safety and independence [37,38,39]. Off-the-shelf gadgets offer specific solutions (e.g., GPS watches) and not an entire platform embedded within inpatient care procedures. For example, GPS devices have been shown to increase safety and independence for PwDs, as reported by family caregivers [37,38,39,40]. However, these devices did not involve alerts or detect indoor locations that would be useful for monitoring within the community (in the early stages of dementia, when PwDs are still going outdoors) or in institutions (moderate-to-severe dementia). In a recent development, remote activity monitors were designed to track multiple residents’ social interaction behaviours based on bed usage (e.g., staying on the bed for too long means the resident is alone, versus both residents not in bed means they are interacting) [41,42,43,44]. Despite the high level of technical advancements, these types of smart environments are designed for unique scenarios, which have no clinical purpose. Current developments cover only a few aspects of activity and behaviours that are possible to monitor in the context of dementia, e.g., in-home tracking of walking speeds and variability in patients with mild cognitive impairments [45].

Ambient sensing has been considered over the last two decades as an approach to patient monitoring; however, current practice does not involve ambient technology to monitor potentially dangerous encounters between PwDs and other persons in an LCTH context. Most literature focusing on ambient sensing to monitor cognitive health of behavioural disturbances have reported on technology development and feasibility studies with older adults and PwDs [46,47,48,49]. The literature has traditionally examined routine-action monitoring such as posture analysis [50,51,52,53,54,55,56] and safety monitoring [57], fall detection (e.g., [58,59,60,61,62,63], indoor localization (e.g., [64,65,66], and wandering (e.g., [67]). Healthcare professionals’ perspectives on the use of ambient sensing technologies have been explored in a variety of settings, including at a people’s home [68,69], in laboratories [70,71] and in healthcare institutions [72,73]. In home settings, while healthcare professionals appreciated the use of smart home technology to monitor chronic health conditions [68,69], they have stressed that technology should be functional and reliable for users [68] and that privacy should be guaranteed in order to increase trust and prevent loss of dignity [68]. Stigma, such as making technology for “old” people, has also been reported as a red light by healthcare professionals. Studies aimed at assessing prototypes of future home-based solutions also encourage the use of technology to monitor chronic health conditions. For example, nurses have shown high levels of acceptance of the design of environments for ageing by the The Lower Saxony Research Network Design of Environments for Ageing and feel technology will assist with medical care, reminders, documentation, fall detection, and security [70]. The use of video cameras in the home was also well received as an appropriate approach to monitoring PwDs and older adults [71]. Monitoring the activities of daily living in assisted living units [72] was well perceived by healthcare professionals. Technology is considered feasible, accessible and easy to use and useful for early detection of health issues, behavioural monitoring, intervention planning, and coordination of care [72]. Healthcare professionals also appreciated the value of sensor-based technology and felt sensor-based technology would improve resident safety, comfort and well-being [67]. Health care professionals have shown particular interest in using surveillance technology to monitor falls and have mentioned that video replay is useful for grading the severity of injury and for screening patients for external referrals to the emergency room [73]. This paper presents a detector of behavioural disturbances (DBD) designed to monitor PwDs residing in LTCH and examines the perspectives of healthcare professionals towards its implementation in a tertiary care facility (Riverview Health Centre, Winnipeg, Canada).

### 1.2. Description of the Detector of Behavioural Disturbances

**Figure 1:**
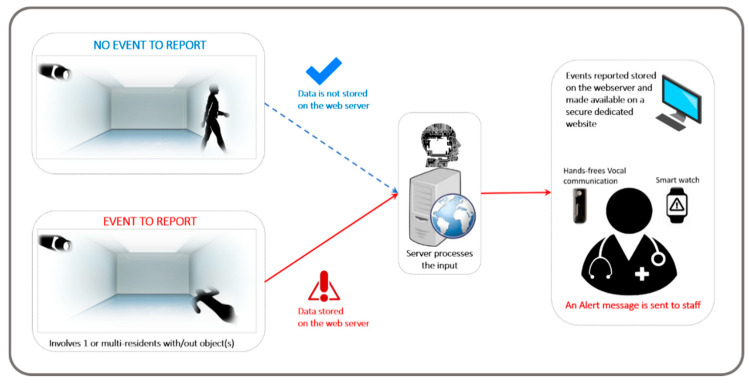
Graphical representation of the internal logic of the behavioural disturbances detector.

The first e-prototype of the DBD was developed in the Rehabilitation Technologies Lab at the University of Manitoba (Figure 1). The DBD is designed to continuously monitor the activity and behaviours of PwDs living in long-term care homes (LTCHs) and send alerts to care professionals when required. A brief description, time and location of the event is included in the message sent to the healthcare professional (e.g., fall/8:00 pm/room 9). East et al. (2020) have described the design and development of the platform [74].

The e-prototype of the DBD has been preliminarily validated in laboratory conditions and is in need of input from its potential users, namely, the LTCH staff. Therefore, the adoption of such technology needs to be explored. The technology acceptance model (TAM2) [75,76] has been identified as an analytical model in this study. TAM2 is the most common paradigm relating to the adoption of digital technologies. The TAM2 model indicates that the behavioural intention to use a technology precedes its use, which will lead to acceptance and adoption. TAM2 was extended to illustrate factors affecting behavioural intent, including success expectations, commitment expectations, social impact, and facilitating circumstances, hedonic motivation, size, importance and habit in the Unified Technology Acceptance and Use of Technology theory [77].

The purpose of the present study is to evaluate the potential of implementing a DBD in LTCHs by mapping the perceptions of healthcare professionals toward this technology. The first objective of this study is to discuss the functionalities of the DBD to evaluate if they meet the clinical and safety needs of daily practice. The second objective is to examine the perspectives of healthcare professionals on the acceptance of the DBD as comprehensive technology for monitoring PwDs living in LTCHs.

## 2. Materials and Methods

### 2.1. Study Design

A focus group (FG) was conducted in September 2019 at Riverview Health Centre (Winnipeg, MB, Canada) in order to explore the potential of implementing a DBD to monitor PwDs living in LTCHs.

### 2.2. Focus Group Participants

Nine persons took part in the FG (Table 1), among which eight were healthcare professionals employed at Riverview Health Centre with more than 2 years of experience with PwDs. All participants were female. Participants’ professions included social worker (1), nurse (2), health care aide (1), rehabilitation assistant (1), occupational therapist (1), recreational therapist (1) and physiotherapist (1). The ninth participant was the partner of a PwD living in the Alzheimer Centre of Excellence (ACE) of the Riverview Health Centre for the past two months. The latter showed interest in the study and contacted the study coordinator to share her perspective during the focus group. Pseudonyms have been used throughout this document.

### 2.3. Data Collection

**Figure 2:**
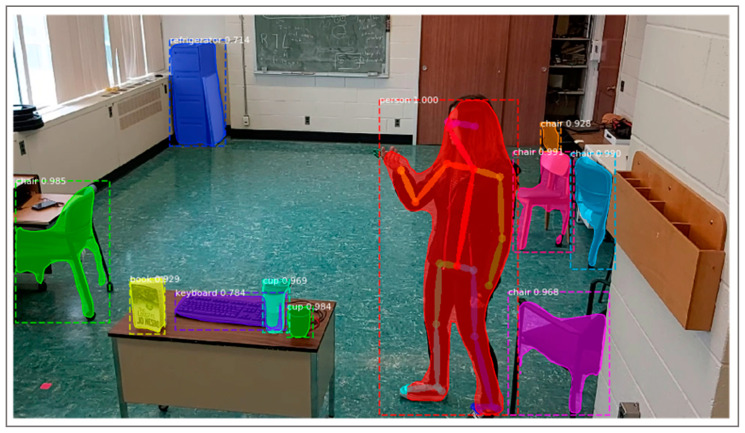
Example of the processing of pose detection presented to the focus group participants.

FG methodology followed general advice for FG management (e.g., [78,79,80,81]). The FG interview schedule had the following general steps [78]: (1) Welcome and introduction, assurance of confidentiality and obtainment of background information, (2) an overview of the topic, (3) statement of the ground rules of the FG, (4) the questions following an FG guide and brainstorming, and (5) summary and wrap-up. Step 2 (overview of the topic) was supported by a 15-min presentation of the technology, mainly supported by a graphical representation of the DBD components and internal logic of the alert system and data storage (Figure 1). Figure 1 shows the system interaction diagram of the DBD. The system actively monitors the patients’ living environment in real-time using a set of depth cameras, discarding the captured data when it does not indicate a critical situation and saving those which are critical without breaching the privacy of the patient (Figure 2).

When a critical situation is realized, the system will instantly notify the care facility on their preferred device, ranging from desktop PCs, vocal systems, smartwatches, or even communication systems already used in health facilities. Step 4 (FG guide and brainstorming) is based on the technology acceptance model (TAM2) [75]. According to the technology acceptance model (TAM) [75], the level of acceptance of technology depends mainly on the user’s perceived ease of use and perceived usefulness [75,82,83,84]. The FG was conducted by two moderators and lasted 1.5 h. The FG was audio-recorded and transcribed verbatim.

Figure 2 shows the capability of the behavioural disturbances detector to detect persons and objects in the environment. Only stick figure coordinates will be stored on the webserver when the event is subject to alert in order to avoid any potential cyberattacks. Access to video/image contents will not be stored on the webserver. Algorithms are able to translate the coordinates into an event (e.g., in this figure, throwing scissors).

### 2.4. Analytical Plan and Reporting of Results

FG content was analyzed using a thematic analysis approach following deductive coding [85]. Themes emerging from the thematic analysis were then clustered to correspond to the TAM2 framework [75]. According to TAM2, the level of acceptance of technology depends mainly on the user’s perceived ease of use and perceived usefulness [52,75,83,84]. Therefore, the aim of the analyses is to evaluate the care professionals’ perspectives on the acceptance of the DBD [75], which determines their intention to implement the DBD as a monitoring intervention in their institution and their potential usage behaviour [76]. Acceptance is seen in the literature as a barrier or facilitator to the successful adoption and implementation of a new system [75].

## 3. Results

Three themes emerged from the thematic analysis of the perspectives of care professionals using the DBD to monitor PwDs in LTCHs: (A) the ability of the DBD to detect relevant behavioural disturbances that are typical of PwDs; (B) the characteristics of the DBD and clinical needs and preferences; (C) the integration of the DBD into daily routines. Each theme is supported by subthemes, as exemplified by FG participants.

### 3.1. Ability of the DBD to Detect Relevant Dementia-Related Behavioural Disturbances

Participants shared a range of behavioural disturbances PwDs often engaged in with other individuals such as staff and other patients, as well as hazardous behaviours they imposed on themselves. As Brenda, a physiotherapist, communicated, the behaviours of PwDs can be compared to that of a child:

“*I hate to put this in words, but think of an elderly PwD…any kind as being a child again. Would you leave your child with, without being watched at any given time? Or any place?...Of course not, but that’s what happens. And when in a nursing facility, there isn’t the staffing to have the one-on-one, which would be appropriate, as you would with a child*.”

In order to delimit the capabilities of the DBD, FG participants were presented with a list of behavioural disturbances among the list of behaviours the first e-prototype of the DBD was designed for and were asked to comment on the list or add behaviours as needed. Two categories of behavioural disturbances emanated from this discussion:

- Behaviours between persons (n = 6): “*fighting another person”, “slapping another person”, “pushing another person”, “kicking another person”, “stabbing another person”*, and *“punching another person”*.

Participants reported a range of behavioural disturbances PwDs engaged in with other individuals; most frequently, the reported altercations will occur between residents. As Mia, a nurse, stated, *“if you put all the residents together…its, its. There’s going to be altercations just by the nature of personalities”*. Mia further signified, *“quite often, residents will trigger each other. There’s just something about that other resident”*. This type of behaviour between residents had also been observed by Emma, a social worker, who detailed *“one resident may slap another resident, push another resident…”* in addition to hitting and punching, to which Charlotte, a rehabilitation assistant, added that residents have also kicked and stabbed one another.

- Individual behaviours (n = 8): *“walking without a walker”, “falling”, “throwing items”,“ grabbing items off walls”, “walking into another person’s room”, “biting themselves”, “putting things in their mouths*, and *“scratching themselves”*.

Participants also identified common behaviours PwDs engaged in by themselves, which comprised of actions occurring as a result of the PwD engaging in the activities of daily living. The most common hazardous behaviour participants reported PwDs engaging in was falling. As Brenda, a physiotherapist, described, *“as in accidents, it usually becomes closest to the stop sign, getting in and out of bed, on and off the toilet and those things people generally want to do on their own”*. Jessica, a recreational therapist, added that falls also occur as a result of PwDS not using their walker. Jessica explained, *“if there’s a way to detect that the person is supposed to have a walker and they don’t, cause that will prevent a fall”*.

In addition to falls, participants also conveyed actions PwDS engage in can also be aggressive in nature. As Emma, a social worker, and Evelyn, an occupational therapist, pointed out, PwDs often bite themselves, put things in their mouth, scratch themselves, and even attempt to pull things off the walls. However, at times, the activities PwDs engage in may not be aggressive in nature but are still concerning and/or hazardous for staff members. For instance, Mia, a nurse, explained, *“just you know they’re impulsive because they’re doing things they are not capable of doing, deciding they’re going to climb on the table or something like that”*. This demonstrates that even though some of the behaviours PwDs engage may not be considered aggressive, there is still merit in alerting staff about those behaviours.

### 3.2. Characteristics of the DBD and Clinical Needs and Preferences

Healthcare professionals partaking in this FG notified us of the need for this technology in their work environment. Evelyn, an occupational therapist, noted that due to the inability of the healthcare system to hire more care professionals, technology could help fill this void:

“*Yeah, I think best case scenario, obviously, we all want more bodies on the floor to help with, you know, the work load. It’s a lot for the staff, so we want some more staff on the floor uhm…but because that’s not really a feasible option these days, it seems like any kind of technology that could help*.”

#### 3.2.1. Benefits of the DBD

- Safety and quicker response time

Participants uncovered multiple potential benefits of having the technology to detect PwD behavioural disturbances. Participants indicated prevention of hazardous events, increased safety and quicker response times as potential benefits of this technology. Charlotte, a rehabilitation assistant, suggested it could even assist nurses and healthcare aides in completing their tasks more efficiently:

“*It would give maybe nursing and healthcare aides a little bit of peace of mind too that they can do their job better because they’re not constantly having to leave while they’re handing out meds…I think it would be a little more efficient*.”

- The DBD as a recording device

One unexpected benefit Evelyn, an occupational therapist, proposed to the group is using this technology as a monitoring device to detect and record certain hazardous behaviours such as falls that have occurred:

“*If it’s always going to be real-time or something that’s recorded, then that might be more helpful for OT to kind of go back and look back at what was the factors…So it would help to piece together…potentially how that fall happened, which would…might be helpful for occupational therapists or physiotherapists*.”

Support for being able to rewind specific events using this technology also came from Brenda, a physiotherapist, who indicated, *“[sometimes] they might, as we said, come out with that hit and we don’t know [what happened], did someone else hit them, did they fall, did they bump their head. It would give us a map of who”.*

In contrast, participants also saw the benefit of using this technology in real-time, specifically during night shifts, as Mia, a nurse, recounted:

“*I’ve had incidents too, where someone’s been injured by another client, and they’re not found because that person left the room. Or someone’s fallen, and so it’s not someone that you know is high risk and they’ve fallen in the bathroom. This would make me be able to go to them right away than them lying there. Especially if it’s evenings or nights and you’re all busy, and you’re making 1-h checks, someone could be lying on the floor*.”

Consequently, this led to the potential dual use of the DBD by having it act as both a recording device to retrospectively analyze past hazardous events and as a live tool to increase response times in the evening and to proactively prevent potential hazardous behaviours from taking place. Mia, a nurse, expressed that having technology such as this may also help increase the level of trust between staff and the family members of PwDs by stating:

“*I think it would help build the trust level between staff and families because we could be more transparent with what actually occurred. I think that would increase our trusting*.”

Emma, a social worker, conveyed that families had even requested cameras to monitor their loved ones, *“we’ve actually…sorry…we’ve actually had lots of families that have asked to get cameras in the room, but because of PHIA, we always say no, so I don’t think you’d get any big explanation”*.

- Support for family members

As the partner of a PwD, Olivia (partner of a resident) believed other family members would also be supportive of having technology monitor PwD behavioural disturbances. Olivia stated:

“*Well, the families would be more relieved knowing…more at peace when they go home. Knowing that there are cameras… Like if something happens in the room, it’s going to help, I guess, the family to know that somebody is coming if an incident happens*.”

Prior to the FG, Olivia had expressed feeling safe in this setting and being able to voice her thoughts.

#### 3.2.2. Limitations of the DBD

- Cost

One of the limitations brought up by participants was the cost. As previously mentioned, Evelyn had suggested that the technology could be used as a means to fill the void of not being able to hire more healthcare professionals. However, in implementing technology, the cost may impose a limitation. This was expressed by Emma, a social worker, who indicated:

“*Cost is always a challenge these days, but purchasing the equipment, paying for things like training. Yeah, I mean the reality of the climate these days is cost is always a barrier and an issue*.”

- Integration into the workflow

Participants also presented concerns surrounding how the technology would be used and integrated into the roles of healthcare professionals’ responsibilities. Evelyn, an occupational therapist, articulated her concerns about individuals relying heavily on the technology, indicating:

“*You don’t want, really, people sitting there watching the screen when ideally we should be engaging with the residents, right*.”

Subsequently, Evelyn added that in order to prevent the misuse of such technology, clear expectations of how the technology should be used ought to be communicated to the workers:

“*I think communication of expectations of how it should be used as a resource or how much time should be spent watching it versus being out there, that kind of balance. So communicating how to use the tool effectively, I guess*.”

- Potential for behaviour change

Brenda, a physiotherapist, suggested that technology may not be the right solution for responding to hazardous incidents, as it is the response time of staff members that has a greater implication for PwDs:

“*A person can only run as far as they can, you can have all the technology in the world, but it’s that person getting to that incident in an amount of time down the hall and that’s staffing, so…as you said…Uhm you know you can see it on camera, but how fast can you get them to…can you get them from A to B?*”

This sentiment, shared by Brenda, received varied reactions from other FG participants; however, alternative uses of the technology suggested by other participants were seen as potential benefits of the technology.

### 3.3. Integration of the DBD into Daily Routines

Discussions surrounding how to use the DBD for monitoring behavioural disturbances and hazardous behaviours among PwD gave rise to three subthemes, namely, the location of the said technology, modes of communication and which professions should have access to the DBD at Riverview Health Centre. In discussing these elements of the implementation process, there was a strong consensus among the FG participants that no matter how this technology is implemented, it should be easy for staff and integrated with the technology already being used at the facility. As Emma, a social worker, put forward:

“*You want to make, like, it easier for staff, not add another thing… Like something that’s already linking to what we have versus kind of adding on*.”

#### 3.3.1. Location

FG participants expressed that the blind spots in the ACE would be beneficial spots to place cameras. Emma, a social worker, indicated, *“the blind spots, like kind of around the hallways, corners; like by the nursing station you usually can see, but it’s usually like all the little pods”.* This view was supported by Mia, who disclosed, *“quite often, we don’t know who caused the injury, cause lots of times, round corners, you don’t see a punch or something like that”*. In addition to the blind spots near the nurses’ station, Jessica noted the blind spots in the pavilion, *“it would probably be like worsened in this area, like we can’t see by where the car is and where the little gas station and the table and the sinks at the… so we can’t see by the end”.*

Participants also suggested placing the cameras in spaces PwDs experience falls, which often occur in spaces they engage in activities of daily living. The rooms of PwDs were often mentioned as locations of falls. For instance, Mia, a nurse, stated, *“...quite often a fall will be from something out of bed”*. In addition, Brenda, a physiotherapist, highlighted that the cameras should also be placed in additional locations where falls occur, which was often *“when a person tries to go on and off the toilet themselves”.* Both cases verbalized by FG participants demonstrate a need for the placement of cameras not only in the rooms of PwDs, but also in their washrooms. Olivia, the partner of a PwD, also expressed her support for placing cameras in the rooms and washrooms of PwDs:

“*...definitely, because it does escalate in the room with my partner, and I…I had to back off or he was ready to hit me, but, like you say, he…that, that wasn’t his personality before, so you really have to be patient with them and realize where they’re coming from and what’s happened to them, but not all family members know that... They can’t understand and it’s very difficult so to have a camera in the room or something by us would just [be] great*.”

#### 3.3.2. Modes of Communication

In order to communicate behavioural disturbances from the DBD to staff members at Riverview Health Centre, there was a strong consensus that the selected mode of communication be integrated with technology already being used at the facility. Participants discussed one main piece of technology that was accessible by all staff members at Riverview Health Centre, that being a “smart pager”. The current system is a communication device that allows staff members to contact one another when needed in a fast and secure fashion. Emma, a social worker, noted that it would be beneficial and practical *“if you can develop the technology that connects to the smart pager because everybody here wears that”.* In addition to the smart pager, participants mentioned iPads were located near the door of the room of PwDs to display pictures of their family members. Emma revealed, *“we have iPads on every room. If somehow they can connect the technology to like those screens blink for like a couple seconds and then it goes back to the pictures”*. Following these suggestions, Emma, a social worker, and Mia, an occupational therapist, suggested ‘r*ather the smart pager blinks and then the, one of the tablets…turns purple or like red or something”.* This integration of the newly proposed DBD with existing technology would not only make its implementation easier for staff members by not adding new technology to their day-to-day routine but also assist in smarter spending for LCTHs.

#### 3.3.3. Professions

It was apparent among FG participants that in order for the DBD to be implemented in Riverview Health Centre, all health care professionals in the ACE would require access. As Emma, a social worker, noted, *“all of us allied health are more common, like on a consult base, so we’re not in the ACE all the time. It’s kind of as needed. We cover multiple units, so it’s more nursing and healthcare aides who are there all the time”.* This suggests there is a more immediate need for nurses and healthcare aides in the ACE to have first access to this technology when it is implemented, as they are often first responders to hazardous behaviours PwDs engage in. As Emma illustrated:

“*You’d need the whole floor like the nurses and healthcare aides who are there all the time. I think all of them would have to be able to have like that quick time access cause a nurse might be on rounds and it’s the aides on the floor or a nurse is in one room and like I think it has to be everybody, you can’t just assign one just by the nature of what happens day-to-day… It’s your aides and nurses that really need that information the most cause they are like very front-line and would respond first*.”

Nonetheless, despite nurses and healthcare aides being cited as professions in most need of accessing this technology, allied healthcare professionals also saw the potential benefits of using the DBD in their respective roles.

## 4. Discussion

The first objective of this study was to evaluate the match between the functionalities of the DBD and healthcare providers’ daily clinical and safety needs. The healthcare professionals’ opinions confirmed that the DBD is an appropriate platform that meets their daily needs in terms of detecting and alerting them about hazardous behaviours or personal and interpersonal behavioural disturbances. As FG participants mentioned, hazardous behaviours or personal and interpersonal behavioural disturbances are indeed inevitable in PwDs, given the effect of their disease and emotional state on their behaviour [3,4,5]. The participants unanimously supported the idea behind using the DBD as zero-effort technology to support the care provided by staff members to PwDs and to support the safety of everyone in the ACE, in accordance with the literature on patient telemonitoring in long-term care facilities [67,72,73]. Indeed, such a platform is necessary to allow staff access to information that they cannot otherwise access. For instance, when discussing the functionalities of the DBD in daily practice, Emma, a social worker, stated, *“this could be great”.* Residents of the pavilion dedicated to PwDs in Riverview Health Centre actually wear a bracelet that alerts staff when a patient leaves the pavilion. However, Emma commented on the DBD that *“this* *simple idea is surprisingly unavailable for our healthcare facilities, even though it is very much needed”.* This shows that an ambient solution like the DBD adds to existing wearables, e.g., the bracelet, which has also been shown to increase PwD safety and independence, as reported by family members [37,40] and healthcare professionals [67,86]. Our FG participants expressed high “perceived usefulness” of the DBD as it covers behavioural disturbances in blind spots (e.g., hallways, corners, entrance by the nurses’ station), the pavilion in general, and even in the resident’s room and washroom, which was supported by a family member involved in the current study and the literature [72,86]. Participants clearly established that all staff members working in the ACE should have access to this technology (“subjective norms”) and that if there is a need to prioritize which professions should have access to this technology, nurses and healthcare aides should take priority as they are more present in the front line and closely interact with the patients on a daily basis. While the literature on procedures related to long-term care use of ambient sensing systems is rare, the literature has stressed the need for appropriate organizational changes to accompany the adoption of new systems [39,87,88,89]. The pragmatic discussion within the FG members about “who should use the DBD and how” highlights high “intention to use” the DBD, namely, organizing who will first receive the alert message and how the information will be sent to the closest and more likely to be available staff member. Of importance to note, the prioritization of which professions should first have access to this technology will vary among each institution. However, whatever the organization of workflow, the DBD is considered very promising technology and has been clearly described by Isabella and Mia as “easy to use’ and “easy to integrate into the daily routine” (“ease of use”) and as a facilitator of communication between staff, to the benefit of timely and appropriate care to the patient.

The second objective of this study was to map healthcare professionals’ perspectives on the acceptance of the DBD as comprehensive technology to monitor PwD living in LTCHs. Staff members partaking in our FG conveyed support for the implementation of this technology in their work environment. “Perceived usefulness” and “ease of use” were high according to the opinions of the FG participants, as illustrated by Mia, a nurse, who indicated the DBD was *“a brilliant idea”*. However, our results revealed that the adoption of the DBD would need to come with brilliant updates of the working procedures related to safety and interprofessional collaboration (i.e., in terms of who first receives the alert message and dispatches the information to the colleague who is most likely to intervene in a timely manner). As discussed in the literature [23], such an update of working procedures will promote a better approach (behavioural change and interprofessional collaboration) on the basis of patient needs and the prioritisation of all tasks in the pavilion. Interprofessional collaboration was not discussed thoroughly and very explicitly during the FG. However, FG participants mentioned unanimously that adjusting working habits to the technology should not be a major barrier and suggested an initial training session prior to implementing the DBD in daily routines would increase the standardization of its use (“experience”, “relevance” and “ease of use”). This finding adds a pragmatic recommendation (i.e., staff training) to existing “broad” recommendations in terms of organizational change within healthcare institutions [39,87,88,89]. Although FG participants seemed supportive of implementing the DBD, they also acknowledged potential concerns about privacy, which are expected and quite common in the field. Our FG participants concluded that privacy is a technical process and that the technical protection of privacy and the provision of appropriate operating procedures will provide the green light for the implementation of the DBD [39,68,88,89]. Indeed, patient’s information is not recorded at all in the DBD platform. It is only the location, time, nature of the event and the number of persons involved in the event that are reported to the staff.

Cost has also been reported as a barrier to the adoption of new technologies, but the FG participants agreed that this was more about budget allocation and leadership direction. To our knowledge, there is currently no literature on the economic evaluation or cost of implementing new technologies such as the DBD within healthcare facilities; therefore, further research is needed in these areas. FG participants also had varied opinions on how the quality of the interactions between PwDs and staff members could change after the DBD has been implemented. Some participants thought that the DBD could cause less human interaction due to the monitoring of the alert system or the screens, which would reduce the overall quality of interaction (e.g., less frequent PwD checks), while other participants felt it would allow them to monitor more PwDs at one time, thus helping them with their daily tasks. Potential behavioural changes for clinicians using the DBD require further investigation postimplementation.

*Summary of the benefits of implementing the DBD in an LTCH, learning from discussions with members of the ACE team*:

- Allows nurses and healthcare aides to be more efficient in performing their daily tasks;
- Increases response time to hazardous events;
- Enables prevention of a hazardous event (being able to intervene before a more hazardous event takes place);
- Increases safety for staff (being able to go back and see what exactly happened in the event that staff is accused of malpractice);
- Using this device as a recording would help allied healthcare professionals go back in time and piece together how a fall or another hazardous event occurred;
- Increases the trust level between staff and family members;
- Allows families to feel more at peace when they leave.

## 5. Limitations

Outcomes reflect the opinion of a group of healthcare professionals from one long-term care facility. Therefore, long-term care facilities interested in this technology should be aware of the fact that the level of acceptance of the DBD described in this paper may slightly differ according to the setting. These preliminary results call for a larger study, including a variety of stakeholder profiles. The inclusion of male healthcare professionals would also add a perspective that would not have been covered by a female-only group.

## 6. Conclusions

This paper presents the acceptance of a DBD designed to monitor PwD living in LTCHs. The first e-prototype of the DBD was presented to care professionals of the ACE, who were asked to share their experiences and needs with each other, generate and codevelop ideas about the potential implementation of the DBD, and explore potential issues of shared importance. The DBD is a comprehensive platform that monitors PwD behavioural disturbances in LTCHs and sends alert messages to healthcare professionals when needed. The aim of this study was to evaluate the potential of implementing the DBD in LTCHs by mapping health professionals’ insights into the DBD through a focus group. Healthcare professionals involved in this study noted a range of noteworthy behavioural disturbances and hazardous behaviours PwDs engage in that the DBD could detect, such as hitting other residents, walking without a walker or wandering into another resident’s room in the same pavilion. Multiple potential benefits of having this technology emanated from this study, including increasing the efficiency in completing daily tasks such as handing out medication, increasing the level of trust between family members and healthcare professionals and using this technology as a recording device to compile meaningful information and reconstruct how certain adverse events occurred. This recording feature could be used for legal proceedings if staff are accused of malpractice. It could also be used for staff training purposes. In recognizing the potential benefits of this technology, it is important to be mindful of the potential cost of implementing such a device in healthcare facilities such as Riverview Health Centre and ensure that expectations of use are effectively communicated to staff and families, should the technology be implemented. This study provides food for thought about the requirements of smart long-term care facilities that can be characterized as follows: data-driven patient centeredness, digital networking infrastructure, interconnected staff, cognitively stimulated and connected residents, and informed and proactive families. Although we do not expect many long-term care facilities to embrace digital health in the coming years, we expect the majority to begin gradually, with smaller solutions based on their priorities.

## Figures and Tables

**Table 1:** Focus group participants’ characteristics.

Pseudonym	Role	Experience in the Dementia Unit
Olivia	Partner of a Resident	The resident has been admitted to the unit for 8 weeks
Emma	Social Worker	20 years in the dementia unit and 2 years on the special needs and special needs behaviour unit
Ava	Nurse	Over 2 years and been to the special needs unit for over a year
Isabella	Health Care Aide	22 years
Charlotte	Rehabilitation Assistant	14 years working with geriatrics
Mia	Registered Nurse	16.5 years on the dementia unit
Evelyn	Occupational Therapist	2 years in the dementia unit and 1 year in a personal care home
Jessica	Recreation	2 years in the dementia unit and 15 years in a personal care home
Brenda	Geriatric Care	20 years in geriatric care and 5 years in the dementia unit

## Data Availability

Not applicable.
